# Self-evaluation and professional status as predictors of burnout among nurses in Jordan

**DOI:** 10.1371/journal.pone.0213935

**Published:** 2019-03-22

**Authors:** Othman A. Alfuqaha, Mahmoud Y. Alkawareek, Hussein S. Alsharah

**Affiliations:** 1 School of Educational Sciences, The University of Jordan, Amman, Jordan; 2 Jordan University Hospital, Amman, Jordan; 3 School of Pharmacy, The University of Jordan, Amman, Jordan; Groupe ESC Dijon Bourgogne, FRANCE

## Abstract

The aim of this study is to evaluate the contribution of self-evaluation, professional status and several demographic factors in predicting burnout among nurses in Jordan. This study was performed on a stratified convenience sample of 350 nurses from 6 hospitals. Modified versions of burnout inventory, self-evaluation scale, and professional status scale were developed, validated and used in this study. Burnout, self-evaluation, and professional status are all found to be at moderate levels. Furthermore, self-evaluation and professional status are found to be important predictors of burnout. On the other hand, although type of hospital and educational level are found to be associated with the level of burnout, overall none of the studied demographic factors was found to be a significant predictor of burnout among nurses in Jordan.

## Introduction

Nurses usually face prolonged exposure to stressors in work environment and hence they are particularly prone to the problem of burnout which may result in decreased quality of care and increased job turnover [1]. Psychological burnout is comprised of three core dimensions which are emotional exhaustion, depersonalization, and reduced personal accomplishment. Nurses in Jordan, as in other countries, generally suffer higher levels of burnout compared to other professions [2, 3].

Self-evaluation (SE) is an essential contributor to reduced personal accomplishment which involves low self-perception, sense of failure, low self-esteem, and inability to achieve development and productivity at work [4]. SE can provide a general overview of the nursing performance in hospitals. Nowadays, core self-evaluation (CSE) model is an emerging branch of organizational psychology that constitutes of four core components, namely locus of control, neuroticism, generalized self-efficacy, and self-esteem. This model indicates that a high level of self-evaluation leads to reduced level of burnout in addition to higher levels of engagement, self-confidence and satisfaction at work [5]. A study on USA nurses also showed that high self-evaluation results in less emotional exhaustion, less depersonalization, and higher self-efficacy [6]. Another study among Chinese nurses found that self-evaluation was significantly associated with burnout, job satisfaction, and organizational commitment [7].

Professional status (PS) is the perceived image of one’s profession compared to other professions or other people [8]. Nurses who have lower professional status may suffer from certain physical and psychological problems. Such nurses have been shown to have high blood pressure and increased heart rate [9]. They were also found to suffer from feeling of worthlessness, dissatisfaction, low self-esteem, and poor social status leading to higher levels of burnout [9]. Low professional status and the associated burnout may also raise an intention to leave the nursing professional together [10]. A study carried out in Canada found that many nurses who emigrated there from the Middle East did so mainly because they had low perceived levels of professional and social status in their home countries [11].

Many studies have associated burnout with many demographic factors such as gender, age, marital status, type of hospital, work experience, work position, and educational level [12, 13]. For instance, male nurses in Japan were shown to experience signs of depersonalization more than female nurses, while female nurses were shown to experience signs of emotional exhaustion more than their male counterparts [14]. Single nurses were found to suffer from burnout more than married nurses in both Taiwan and Nigeria [15, 16] but the opposite was found in Lebanon and Rwanda [17, 18]. A study in India, showed that nurses working in public hospitals exhibit higher levels of burnout than nurses working in private hospitals [19], while the opposite was observed in Sweden [20]. In 2016, a review of 203 scientific studies was performed and found that nurses in the intensive care unit (ICU) suffered from burnout significantly more than those in other hospital’s wards [21]. Whereas another study indicated that nurses working in the emergency room had the highest level of burnout [22]. Moreover, high educational level among nurses in Iran were found to be the highest level of burnout among other degrees [23], while the opposite was observed in 2014 [24] that indicated a lowest educational level (diploma) were found to be the highest level of burnout among nurses because of role conflict.

In order to properly address and tackle the problem of burnout among nurses, it is important to understand the factors that lead to its development. Although there are many studies discussing this issue around the world, there are only a limited number of such studies in Jordan and, to the best of our knowledge, none of them was directly involved in studying self-evaluation and professional status as potential predictors of burnout among nurses in Jordan; which is the main aim of this study. In addition to self-evaluation and professional status, several demographic factors including gender, marital status, type of hospital, experience, work position, and educational level were also investigated as part of this study in order to evaluate their association with burnout and whether any of them can be a predictor of burnout among nurses in Jordan.

## Methodology

### Study questions

1. What is the level of burnout among nurses in Jordan?
2. What is the level of self-evaluation among nurses in Jordan?
3. What is the perceived level of professional status among nurses in Jordan?
4. What is the contribution of self-evaluation, professional status, gender, marital status, type of hospital, experience, work position, and educational level in predicting psychological burnout among nurses in Jordan?

### Study design

This study adopts the descriptive cross sectional design and predictive approach to evaluate the contribution of self-evaluation, professional status, gender, marital status, type of hospital, experience, work position, and educational level in predicting psychological burnout among nurses in Jordan.

### Study population and sampling procedure

The study population consisted of 2500 nurses working in 6 different hospitals, two public and four private hospitals. These hospitals are located in two major cities in Jordan: Amman (The capital of Jordan) and Zarqa which were chosen since they have large number of nurses and are readily accessible to the researchers. The sample in this study consisted of 500 nurses and was selected according to the stratified convenience sampling method. Initially, the study population was divided into two major groups according to the type of hospital (i.e. public vs. private). Then, a convenience sample consisting of 20% of the nurses in each major group was taken as detailed in S1 Table. Out of the selected sample, a total of 400 nurses returned the questionnaire indicating a response rate of 80%. Due to missing data, 50 incomplete questionnaires were excluded and hence the analysis in this study was performed on a final number of 350 questionnaires. Relevant ethical guidelines were followed during the distribution of questionnaires in each hospital. The study was conducted between May and July in the year of 2017. The nurses participated voluntary and the researchers distributed the questionnaires during break times of nurses in different shifts. The participants were informed that completing the questionnaire is taken as an informed consent to participate in this study. Table 1 shows the detailed properties of the selected sample with more details presented in S1 Table.

**Table 1:** Properties of study sample (N = 350).

*Variables*	*Descriptive*	*Frequency*	*Percentage (%)*
***Gender***	Male	147	42
	Female	227	64.9
***Marital status***	Single	149	42.6
	Married	201	57.4
***Type of hospital***	Public	203	58
	Private	147	42
***Experience***	1-5Y	163	46.6
	6-10Y	102	29.1
	≥11Y	85	24.3
***Work position***	Surgical Ward	101	28.9
	Medical Ward	82	23.4
	Intensive Care Unit	79	22.6
	Emergency Room	88	25.1
***Educational level***	Diploma	68	19.4
	Bachelor	228	65.1
	Post Graduate	54	15.4

### Research tools

All scales of the study have been described separately as follows:

#### Burnout

Modified Maslach Burnout Inventory (Modified-MBI), as described previously [2], was used to measure the three core dimensions of burnout. Emotional exhaustion (EE), a measure of emotionally overwhelming feelings, loss of energy and tiredness, consisted of 9 items. Depersonalization (DP), a measure of negative views toward patients and loss of idealism, consisted of 7 items. Personal accomplishment (PA), a measure of decreased level of productivity and efficacy, consisted of 9 items. Items of EE and DP were rated on a 5-point Likert-type scale as follows: 1 'Never', 2 'Rarely', 3 'Sometimes', 4 'Frequently', 5 'Always'. Items of PA were also rated on a 5-point Likert-type but in a reversed order as follows: 1 'Always', 2 'Frequently', 3 'Sometimes', 4 'Rarely', 5 'Never'. The average score of the 25 items (obtained from all 3 dimensions) was calculated and used to assign the level of burnout as mild (for average scores of 1.00–2.33), moderate (for average scores of 2.34–3.66) or severe (for average scores of 3.67–5.00). In order to assess validity and reliability of the Modified-MBI used in this study, the scale was applied on 30 nurses outside the study sample and the obtained results were used to calculate the correlation coefficients between dimensions, Cronbach's Alpha, and Test- Retest values as shown in Table 2.

**Table 2:** Correlation coefficients, Cronbach's alpha, and test- retest values for the modified-MBI used in this study.

*Dimension*	*(EE)*	*(DP)*	*(PA)*	*Cronbach's Alpha*	*Test- Retest*
***Emotional Exhaustion (EE)***	-	0.88	0.65	0.82	0.80
***Depersonalization (DP)***	-	-	0.51	0.75	0.84
***Personal accomplishment (PA)***	-	-	-	0.73	0.81
***Total Score***	0.79	0.74	0.72	0.76	0.82

#### Self-evaluation

A Self-Evaluation Scale was developed for the purpose of this study to measure self-evaluation among nurses in Jordan. The scale consisted of 17 items and was developed according to previous studies [25, 26] and modified based on a survey of 10 specialists in educational psychology and measurement in Jordan. Content validity and reliability were evaluated by applying the scale on 30 nurses outside the study sample. Correlation coefficients between items were calculated and were the range of 0.64–0.92, Cronbach's Alpha was 0.72, and Test-Retest value was 0.77.

#### Professional status

A Professional Status Scale was used to measure the perception of professional status among nurses in Jordan. The scale consists of 18 items and was developed according to [27] and modified based on a survey of 10 specialists in educational psychology and measurement in Jordan. Content validity and reliability were evaluated by applying the scale on 30 nurses outside the study sample. Correlation coefficients between items were calculated and were the range of 0.71–0.90, Cronbach's Alpha was 0.82, and Test-Retest value was 0.84.

Items of self-evaluation and professional status were rated on a 4-point Likert-type scale as follows: 1 'Strongly disagree', 2 'Disagree', 3 'Agree', 4 'Strongly Agree' for positive items, while the answer scale was reversed for negative items. The average score of all items in each scale was calculated and used to assign the level of SE or PS as low (for average scores of 1.00–1.99), moderate (for average scores of 2.00–2.99) or high (for average scores of 3.00–4.00).

### Ethical approval

Ethical approvals were obtained from the institutional review boards (IRB) of the Jordan University Hospital (No. 10/2017/5269) and the Ministry of Health to allow the collection of information from the nurses participating in this study.

### Statistical analysis

The Statistical Package for the Social Sciences (SPSS version 22) was used for analysis the study results. Descriptive statistics were used in order to evaluate the levels of burnout, self-evaluation, and the perceived level of professional status. Multiple linear regression analysis (stepwise regression) was performed on self-evaluation, professional status, gender, marital status, type of hospital, experience, work position, and educational level in to find if any of these factors can serve as predictor of burnout. To evaluate the association of each demographic factor with the level of burnout, t-test was used for factors comprising two variables (i.e., gender, marital status, and type of hospital) whereas one-way ANOVA test was used for factors comprising more than two variables (i.e., experience, work position, and educational level).

## Results and discussion

The main aim of this study is to evaluate the contribution of self-evaluation, professional status, gender, marital status, type of hospital, experience, work position, and educational level in predicting psychological burnout among nurses in Jordan. To answer the study questions one, two, and three; means, standard deviations, and overall levels were measured as illustrated in Table 3. For the detailed results, see S2 Table.

**Table 3:** Mean, standard deviation, and overall level for all variables.

*Variables*	*M*	*SD*	*Level*
***Emotional Exhaustion***	3.29	0.62	Moderate
***Depersonalization***	2.53	0.73	Moderate
***Personal Accomplishment***	2.86	0.60	Moderate
***Burnout***	2.92	0.49	Moderate
***Self-Evaluation***	2.77	0.36	Moderate
***Professional Status***	2.38	0.56	Moderate

### Question One: What is the level of burnout among nurses in Jordan?

Table 3 shows that the mean score of burnout is (2.92 ± 0.49) which indicate that nurses in Jordan suffer a moderate level of burnout. This finding can be explained by the fact that nurses in Jordan are poorly paid, they deal with large number of patients having acute/chronic illness and they face high job demands, lack of resources, work under pressure, lack of social support and conflict with supervisors and colleagues. Added to that is the absence of psychological prevention programs in Jordanian hospitals such as training in stress inoculation and self-control techniques. Table 3 shows that emotional exhaustion exhibits the highest mean score among the three dimensions of burnout. This is important since emotional exhaustion is considered the main core element in burnout and a serious indicator to reach high levels of burnout among nurses. However, all three core elements of burnout (EE, DP, and PA) are found to be at moderate levels among nurses in Jordan. This result is in agreement with several previous studies which reported moderate levels of PA [28], DP [29], and EE [30].

### Question Two: What is the level of self-evaluation among nurses in Jordan?

As shown in Table 3, self-evaluation has a mean score of (2.77 ± 0.36) indicating that the level of self-evaluation among nurses in Jordan is moderate. This result is in parallel with previous studies such as [31, 32] which indicated moderate to low level of self-evaluation among nurses. Such low to moderate levels of self-evaluation can be explained by many nurses reporting their perception of not having much to be proud of, not achieved their career goals, not being evaluated fairly by their supervisors and colleagues, not being properly commended by social media, lack of moral appreciation, working in different shifts leading to lack of opportunities to share families’ moments, and low financial returns compared to their effort in work. Nonetheless, the analysis of results obtained by the modified self-evaluation scale revealed that participating nurses can frequently express their point of view directly, acknowledge their mistakes and take decisions with high self-confidence.

### Question Three: What is the perceived level of professional status among nurses in Jordan?

Table 3 shows that although the mean score of professional status (2.38 ± 0.56) was relatively lower than that of self-evaluation, it still indicates a moderate level. This result can be explained by the observation that nurses in Jordan frequently perceive a lack of authority and power related to their profession and a lack of enough opportunities of promotion at work. Despite that, analysis of the modified professional status scale showed that nurses participating in this study frequently try to change negative views toward the nursing profession and they usually feel that patients and their families show respect and appreciation toward them. This result is in agreement with the study of [9] but in disagreement with some other studies which imply that the nursing profession is taking the right steps to reach a prestigious position [33, 34].

### Question Four: What is the contribution of self-evaluation, professional status, gender, marital status, type of hospital, experience, work position, and educational level in predicting psychological burnout among nurses in Jordan?

Multiple linear regression analysis (i.e., stepwise regression) was performed to find the contribution of self-evaluation, professional status, and several demographic factors including gender, marital status, type of hospital, experience, work position, and educational level in predicting psychological burnout among nurses in Jordan. Results of this analysis are summarized in Table 4 and Table 5.

**Table 4:** Results of multiple linear regression analysis (stepwise regression) for burnout predictors.

*Model*	*R*	*R^2^*	*R change*	*Unstandardized Coefficients*		*t*	*Sig.*
*1^a^*	0.696	0.484	0.484	B	Std. Error	53.382	<0.001**
				4.354	0.082		
				-0.603	0.033	-18.077	<0.001**
*2^b^*	0.732	0.536	0.052	5.067	0.149	36.51	<0.001**
				-0.493	0.039	-13.55	<0.001**
				-0.352	0.061	-6.169	<0.001**

^a^Predictors: (Constant), Professional Status (PS).

^b^Predictors: (Constant), Professional Status, Self-Evaluation(SE).

**Significant at α≤0.05 level.

**Table 5:** Results of the excluded variables according to the multiple linear regression analysis for burnout predictors.

*Model*	*Variables*	*Beta In*	*t*	*Sig.*	*Partial Correlation*
*1*	Gender	.003^b^	.081	.935	.004
	Marital Status	-.035^b^	-.919	.359	-.049
	Type of hospital	.061^b^	1.566	.118	.084
	Experience	-.002^b^	-.052	.959	-.003
	Work position	-.035^b^	-.899	.369	-.048
	Educational level	.085^b^	2.176	.030	.116
	Self-Evaluation	-.260^b^	-6.196	.000	-.316
*2*	Gender	.010^c^	.259	.796	.014
	Marital Status	-.058^c^	-1.592	.112	-.085
	Type of hospital	.054^c^	1.472	.142	.079
	Experience	-.038^c^	-1.021	.308	-.055
	Work position	-.028^c^	-.753	.452	-.040
	Educational level	.068^c^	1.803	.072	.096

b. Predictors in the Model: (Constant), PS.

c. Predictors in the Model: (Constant), PS, SE.

The results presented in Table 4 show that two factors can be considered as predictors of burnout among nurses in Jordan which are professional status and self-evaluation with total variation of 53.6%. Professional status is found to account for 48.4% of the variance in burnout (R^***2***^ = 0.484, *p* <0.001). Similar to this finding, previous studies have also showed that professional status is among the most important predictors of burnout in nurses [35]. Self-evaluation was also found to be a significant predictor of burnout accounting for 5.2% of the variance in burnout (R^***2***^ = 0.052, *p* <0.001). It is noteworthy that other studies have reported other factors as predictors of burnout in nurses, among these factors are engagement, physical/psychological symptoms [15], management issues, interpersonal relationships [36], interpersonal conflict, organizational constraints, and workload [37]. On the other hand, the models obtained by stepwise regression indicate that none of studied demographic factors can be considered as a predictor of burnout as shown in Table 5. However, statistical analysis shows that among these demographic factors, type of hospital, and educational levels are still significantly associated with the level of burnout. For instance, t-test shows that nurses working in private hospitals are found to suffer from burnout more than those working in public hospitals (M _private_ = 3.00, M _public_ = 2.86, *t* = 2.71, *P* = 0.008). As for the educational level, interestingly, one-way ANOVA test shows that the higher the educational level of a nurse the more prone he/she is to burnout (F = 20.37, *P* < 0.001).

Other demographic factors including gender, marital status, experience and work position are not found to be significantly associated with the level of burnout among nurse’s in Jordan. This finding are in agreement with those of many previous studies [38, 39] which showed that demographic factors such as gender, age, marital status, experience, educational level, and having children were not found as predictors of burnout but may facilitate the development of burnout among nurses.

Since the study population was initially stratified into 2 major groups based on the type of hospital (i.e. public vs. private), the multiple linear regression analysis was separately performed on each of these two major groups to see if there is any difference between them in term of the predictors of burnout. As shown in Table 6, professional status and self-evaluation still remain the major predictors of burnout in each of these 2 major groups but with different contribution to variance. In fact, the contribution to variance of professional status and self-evaluation is found to be higher in public hospitals (55.3% and 5.8%, respectively) compared to that in private hospitals (35.6% and 4.0%, respectively). Furthermore, unlike what was found with the overall analysis or with that on private hospitals, marital status is found as an additional predictor of burnout among nurses working in public hospitals but with a mere contribution to variance of 0.8%.

**Table 6:** Predictors of burnout and their contribution to variance according to the type of hospital.

*Predictors*	*Contribution to Variance*		
	Overall	Public Hospitals	Private Hospitals
*Professional Status*	48.4%	55.3%	35.6%
*Self-Evaluation*	5.1%	5.8%	4.0%
*Marital Status*	-	0.8%	-

## Conclusions

Burnout, self-evaluation, and professional status are all found to be at moderate levels among nurses in Jordan. The results of this study show that self-evaluation and professional status are important predictors of burnout. None of the demographic factors investigated in this study seems to be a predictor of burnout. However, type of hospital and educational level are found to be significantly associated with the level of burnout among nurses in Jordan. However, self-evaluation, professional status, and marital status are all found to be important predictors with the level of burnout according to the type of hospital and educational level. Other demographic factors, including gender, marital status, experience, and work position, are not found to be significantly associated with the level of burnout either.

The researchers recommend that nurses, their managers, associated authorities, and society as a whole should cooperate to improve nurses’ self-evaluation and professional status and to provide proper psychological prevention programs in order to mitigate the problem of burnout among nurses in Jordan.

## Supporting information

S1 Table Detailed numbers of study population and selected sample in all hospitals included in the study. (DOCX)

S2 Table The Average score results for each questions in the used scales. (DOCX)
