# Supplementary material for: Self-evaluation and professional status as predictors of burnout among nurses in Jordan
Source: PLoS One. 2019 Mar 22;14(3):e0213935. doi: 10.1371/journal.pone.0213935 (PMC6430417; doi:10.1371/journal.pone.0213935)
Supplement: S1 Table — (DOCX) [file pone.0213935.s001.docx]

**S1 Table. Detailed numbers of study population and selected sample in all hospitals included in the study.**

| **Name of hospital** | **Type of hospital** | **Total nurses in hospital*** | **Sample size (nurses surveyed)** | **Sample Size Percentage** | **Nurses responded** | **Response rate** |
| --- | --- | --- | --- | --- | --- | --- |
| Jordan University Hospital | Public | 800 | 145 | 18.1% | 136 | 93.8% |
| Prince Faisal Hospital | Public | 400 | 95 | 23.8% | 77 | 81.1% |
| Jabal Az Zaytoon Hospital | Private | 250 | 45 | 18.0% | 32 | 71.1% |
| Arab Medical Center | Private | 400 | 75 | 18.8% | 47 | 62.7% |
| Royal Hospital | Private | 200 | 40 | 20.0% | 29 | 72.5% |
| Ibn Al Haytham Hospital | Private | 450 | 100 | 22.2% | 79 | 79.0% |
| **Total** |  | **2500** | **500** | **20.0%** | **400** | **80.0%** |

*Estimated numbers
