# Supplementary material for: Self-evaluation and professional status as predictors of burnout among nurses in Jordan
Source: PLoS One. 2019 Mar 22;14(3):e0213935. doi: 10.1371/journal.pone.0213935 (PMC6430417; doi:10.1371/journal.pone.0213935)
Supplement: S2 Table — (DOCX) [file pone.0213935.s002.docx]

**S2 Table. The Average score results for each questions in the used scales**

| **Modified Burnout Inventory: Emotional Exhaustion (EE)** | **Average** |
| --- | --- |
| Question 1 | 3.85 |
| Question 2 | 3.81 |
| Question 3 | 3.68 |
| Question 4 | 3.31 |
| Question 5 | 3.01 |
| Question 6 | 1.95 |
| Question 7 | 3.93 |
| Question 8 | 3.62 |
| Question 9 | 2.42 |
| **Total Average of EE** | **3.29** |
| **Depersonalization** | **Average** |
| Question 1 | 2.73 |
| Question 2 | 3.00 |
| Question 3 | 1.79 |
| Question 4 | 2.02 |
| Question 5 | 1.72 |
| Question 6 | 2.72 |
| Question 7 | 3.69 |
| **Total Average of DP** | **2.53** |
| **Personal Accomplishment (PA)** | **Average** |
| Question 1 | 2.67 |
| Question 2 | 2.72 |
| Question 3 | 2.73 |
| Question 4 | 3.11 |
| Question 5 | 2.67 |
| Question 6 | 2.74 |
| Question 7 | 3.41 |
| Question 8 | 3.71 |
| Question 9 | 1.96 |
| **Total Average of PA** | **2.86** |
| **Total Burnout** | **2.92** |
| **Modified Self-evaluation Scale (SE)** | **Average** |
| Question 1 | 2.95 |
| Question 2 | 3.00 |
| Question 3 | 2.87 |
| Question 4 | 3.11 |
| Question 5 | 2.59 |
| Question 6 | 2.98 |
| Question 7 | 1.84 |
| Question 8 | 3.26 |
| Question 9 | 1.96 |
| Question 10 | 2.02 |
| Question 11 | 2.52 |
| Question 12 | 2.81 |
| Question 13 | 2.56 |
| Question 14 | 3.35 |
| Question 15 | 3.11 |
| Question 16 | 3.03 |
| Question 17 | 3.12 |
| **Total Average of SE scale** | **2.77** |
| **Modified Professional Status Scale (PS)** | **Average** |
| Question 1 | 1.70 |
| Question 2 | 2.07 |
| Question 3 | 2.65 |
| Question 4 | 2.32 |
| Question 5 | 2.28 |
| Question 6 | 2.79 |
| Question 7 | 2.90 |
| Question 8 | 2.55 |
| Question 9 | 2.86 |
| Question 10 | 1.93 |
| Question 11 | 2.55 |
| Question 12 | 2.64 |
| Question 13 | 2.56 |
| Question14 | 2.48 |
| Question 15 | 1.51 |
| Question 16 | 2.67 |
| Question 17 | 1.90 |
| Question 18 | 2.88 |
| **Total Average of PS scale** | **2.38** |
